# An Exposure-Free Tool for Monitoring Adult Malaria Mosquito Populations

**DOI:** 10.4269/ajtmh.2010.09-0682

**Published:** 2010-09

**Authors:** Nicodem J. Govella, Jason D. Moore, Gerry F. Killeen

**Affiliations:** Ifakara Health Institute, Coordination Office, Dar es Salaam, Tanzania; Disease Control and Vector Biology Unit, London School of Hygiene and Tropical Medicine, London, United Kingdom; Liverpool School of Tropical Medicine, Liverpool, United Kingdom

## Abstract

Catches of *Anopheles gambiae* and *An. arabiensis* with the Ifakara Tent Trap-model B (ITT-B) correlate better with human landing catches than any other method but fail to reduce the proportion of blood-fed mosquito caught, which indicates that users are exposed to bites during collection. An improved C model (ITT-C) was developed and evaluated by comparing with ITT-B in semi-field and full-field conditions in southern Tanzania. The sensitivity of the ITT-C was approximately two times that of the ITT-B: relative rate (95% confidence interval) = 1.92 (1.52–2.42), 1.90 (1.48–2.43), and 2.30 (1.54–3.30) for field populations of *An. arabiensis*, *Culex* spp., and *Mansonia* spp., respectively. The ITT-C caught 73% less blood-fed *An. arabiensis* than the ITT-B in open field experiments and none in semi-field experiments, which confirmed that the C design is a safe trapping method. Validation of ITT-C by comparison with human landing catches and parasitologic measures of human infection status may be necessary to confirm that this design produces consistent and epidemiologically meaningful results.

## Introduction

In the drive to eliminate malaria, mosquito sampling measures are crucial for monitoring changes in human exposure to infections and the effect of vector-control interventions.^1^–^3^ However, existing monitoring methods for adult stages of the *Anopheles* vectors of human malaria all have significant limitations, particularly where densities of malaria-transmitting mosquitoes are low.^4^–^6^ This technology has become increasingly important as malaria control,^7^–^9^ elimination, and eradication^10^ are prioritized by policy makers and significant progress towards lower transmission levels is achieved.^6^,^11^–^15^ Standard entomologic methods often fail to detect^16^ low levels of malaria transmission. Sensitive, scalable, safe, and affordable tools are therefore required to achieve sustained and extensive monitoring of vector populations^4^,^17^ so that control efforts can be managed and optimized.

A new device for sampling malaria vectors in Africa, called the Ifakara Tent Trap-design B (ITT-B), has recently been developed and evaluated as a means to catch malaria vector mosquitoes under conditions of low and high mosquito densities in Tanzania.^18^ The relative sensitivity of ITT-B increased as vector density decreased and exceeded that of human landing catches at the lowest densities^18^ in urban Dar es Salaam. The ITT-B correlated better with human landing catches than any other tested method,^18^ and is remarkably cost-effective under programmatic settings with minimal supervision.^19^ However, ITT-B failed to reduce the proportions of blood-fed mosquito caught relative to that observed in sample obtained by human landing catches.^18^,^19^ The biggest disadvantage of the human landing catch method is the inevitable exposure of human participants to mosquito bites.^2^,^3^,^20^ Thus, ITT-B operators may also have been exposed to mosquito bites.^18^,^19^ Alternatively, these traps may act as resting shelters for freshly fed mosquitoes, and both of these possibilities may cause blood-fed mosquitoes to be caught in the field.

This study reports an evaluation of the mosquito sampling properties of an improved C model of the Ifakara Tent Trap (ITT-C), compared with ITT-B to confirm that this new version is comparably efficacious and successfully prevents operator exposure to mosquito bites.

## Methods

### Field study area.

The field study was conducted in Lupiro village in the Kilombero River Valley in Tanzania. Detailed description of the area is found elsewhere,^21^ and the most recent study showed that *Anopheles arabiensis* is the dominant malaria vector in the area.^18^ This location experiences high *Plasmodium falciparum* malaria transmission with an entomologic inoculation rate exceeding 500 infectious bites per person per year, in spite of high coverage with mainly untreated bed nets.^21^

### Semi-field study system.

The semi-field system or screen house is an enclosed structure with walls of mosquito netting and a polyethylene roof located within the natural ecosystem of the target vector.^22^ The semi-field experiment was carried out within one of three 208 m^2^ chambers of a screen house at the Ifakara Health Institute,^22^ in Kilombero District, south-east Tanzania.^21^

### Sampling methods.

**Figure 1.**
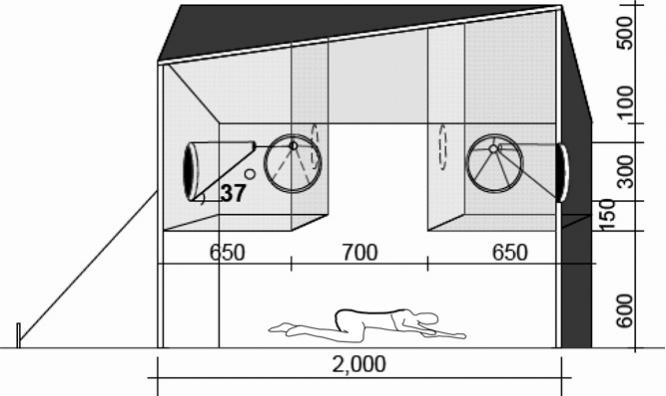
Ifakara Tent Trap-C design. The human occupant is protected from mosquito bites by two rectangular netting panels with the dotted circular point showing the position of the aspirator inlet though a sealable cotton sleeve. Mosquitoes enter through a funnel shaped entrances, each supported by a wire bar with soft caps just outside of the plastic rings, which form the inner small apertures of the funnel end. The three funnel apertures in each trap chamber are drawn tightly together with a three-way elastic band tie, which terminates in these wire bars that hold the ends of the funnels. All dimensions are in millimeters.

The Ifakara B and C traps were the only traps used. Although the ITT-B design has been described in detail,^18^ ITT-C (Figure 1) differs from this earlier prototype in that the netting panel lying between the entry funnels and the bait host is bisected into two compartments within the trap, which are 70 cm apart. This enables a person in the process of collecting mosquitoes to stand up within the trap while protected from mosquito bites. In contrast, the B design requires the opening of the long zipper across the netting panel and aspiration from within the open trap chamber, thereby exposing the operator to bites. Also, there are two long (350 mm) sealable cotton sleeves hanging from each trap chamber to enable operators to safely remove mosquitoes by using mouth aspirators while protected from bites. The two netting chambers, which the baffled entrance funnels lead into, are supported with two string braces to prevent them from sagging or collapsing. This structural feature is important because such sagging of the chambers down upon the occupant would increase the risk of contact with the human bait and thus exposure to mosquito bites. Although the baffled entrance funnels are held by strings suspended from the cross bar in the ITT-B, for the ITT-C they are maintained by wire bars with soft caps just outside of inner small apertures consisting of plastic rings sewn into each entry funnel, all of which are drawn tightly towards each other with a three-way elastic band tie. This feature smooths the entry funnels and probably makes it easier for mosquitoes to enter the trap. For more detail, see the online supplementary material illustrating on how to set up the ITT-C (available at www.ajtmh.org).

### Experimental design: open field.

Four outdoor catching stations were selected approximately 50 meters apart and aligned approximately 100 m from the main rice irrigation area on one side and approximately 15 meters from local houses on other side. Each collector was assigned to and remained associated with a specific sampling station throughout the experiment to control for the effect of differences in individual attractiveness and of a particular station. Two pairs of Ifakara B and C traps were allocated to all four catching stations and a cross-over experimental design was implemented in which each trapping method was exchanged between the two adjacent catching stations on each experimental night. This experiment was conducted for 10 nights (November 18–28, 2008) at a time when there was no rainfall. Mosquitoes were collected by both techniques from 8:00 pm to 7:00 am.

### Experimental design: semi-field system.

Two sampling stations approximately 16 meters apart inside a screen house^23^ were established, and each trap was placed in one of these stations. Two volunteers were recruited and each was assigned to and remained associated with a specific catching station. Traps were exchanged between positions on each experimental night for four nights by using a cross-over experimental design as described above. One hundred starved, insectary-reared, female *An. gambiae* sensu stricto were released from the central release point at 7:00 pm each night and mosquitoes were collected from 7:00 pm to 7:00 am for four nights (November 29 to December 2, 2008).

### Processing of samples.

All anopheline mosquitoes caught were sorted and morphologically identified^24^ directly in the field. The abdominal condition of each female mosquito was classified as unfed, part fed, fully fed, and gravid.^18^ Sub-samples (179 of 344 and 227 of 714) from the ITT-B and ITT-C, respectively, of *An. gambiae* sensu lato (members of this species complex are morphologically indistinguishable^24^) were stored in tubes with desiccated silica for subsequent identification to sibling species level by polymerase chain reaction.^25^

### Data analysis.

#### Mean catch differences between sampling methods.

Although the goal of this study was to test whether the ITT-C is an exposure-free mosquito sampling method, it was also essential to confirm that it is as sensitive as the ITT-B. Using SPSS version 15 software (SPSS Inc., Chicago, IL), we applied generalized estimating equations to quantify the influence of trap design upon mosquito catches by treating station and date as subject and within-subject variables, respectively. The logarithmically transformed catches (log_10_ (x)) for *An. gambiae* s.l., which appeared to be normally distributed, was treated as the dependent variable with an identity link function.

#### Influence of sampling technique upon blood-feeding status of trapped mosquitoes.

Binary logistic regression analysis was used to test for differences in the distribution of abdominal status of mosquitoes from the *An. gambiae* complex caught in the two trap designs. We executed this test by treating abdominal status as a binary outcome, with each mosquito classified as being freshly blood fed (partly or fully) or not (unfed, gravid, semi-gravid), with trap design as an independent categorical factor in the model.^18^,^19^

### Ethical clearance and protection of human participants.

Prior to any field work, research clearance was obtained from the Ifakara Health Institute Ethics Review Committee and the Medical Research Coordination Committee of the National Institute of Medical Research in Tanzania (Reference nos. NIMR/HQ/R.8a/Vol.IX/279 and 324). Informed consent was obtained in writing from all participants before initiation of the study and re-confirmed on each experimental night. These volunteers were screened for malaria parasites by microscopy during recruitment and after finishing the experiment. Those persons who were found to be malaria positive were offered treatment free of charge with artemisinin-lumefantrane (Co-Artem^®^; Roche, Basel, Switzerland) the recommended first-line treatment of malaria in Tanzania.

## Results

### Crude catch sensitivity of the ITT-C relative to the ITT-B.

The crude mean sensitivity of the ITT-C for *An.gambiae* s.l., *Culex* spp., and *Mansonia* spp. relative to ITT-B are summarized in Table 1. The ITT-C consistently sampled approximately twice as many mosquitoes as the ITT-B for all three genera. This difference was significant for *An. gambiae* s.l., the only malaria vector present in sufficient numbers, and for *Culex* spp. and *Mansonia* spp. (Table 2).

### Sibling species composition of *An. gambiae* s.l.

Of 366 successfully amplified specimens of *An. gambiae* s.l. caught in the field experiment, 97% (355) and 3% (11) were *An. arabiensis* and *An. gambiae* sensu stricto, respectively. This finding implies that *An. arabiensis* is the main malaria-transmitting vector in this locality. Therefore, the results presented relating to the *An. gambiae* s.l. species complex overwhelming reflect the response of this particular sibling species to these traps.

### Influence of trap design on the abdominal status distribution.

The ITT-C caught 73% less blood-fed *An. gambiae* s.l. than the ITT-B in the field and none were caught with the ITT-C in the semi-field experiment (Table 3). The observation that six fed specimens were caught with the ITT-B in the semi-field experiment, even though all mosquitoes released were unfed, confirms that mosquitoes feed upon users of the latter design. Although the difference in the proportion of blood-fed mosquitoes between the B and C designs in the semi-field system could not be estimated quantitatively by using binary logistic regression (Table 3), these results nevertheless differed significantly (χ^2^ = 6.78, degrees of freedom = 1, *P* = 0.009).

## Discussion

We demonstrated that modifying the ITT-B improved this prototype beyond our primary target of preventing operator exposure from mosquito bites. The ITT-C sampled twice as many mosquitoes as the ITT-B, which suggests that it may yield mosquito catches more or less equivalent to that of the human landing catches based on previous comparisons of the latter two methods.^18^,^19^ The reasons for a such improved sensitivity with the ITT-C is not obvious but might be explained by increased airflow^26^ caused by the 700-mm gap between the two netted chambers. The use of the elastic band tie, which tightly extends and smooths out the entry funnels, might also have contributed to this improved efficiency because it may make it easier for mosquitoes to enter the trap.

The high proportion of blood-fed mosquitoes caught with the ITT-B matches observations in previous studies.^18^,^19^ The observation that this occurred even in a semi-field enclosure into which only unfed mosquitoes were introduced confirms that persons using this trap are exposed to mosquito bites. This exposure most likely occurs during removal of the mosquitoes because of the need to open the long zipper bisecting the protective netting panel of the B design, as has been reported by field workers in previous evaluations.^19^ The observation that some fully and partially blood-fed mosquitoes from the field are trapped by the ITT-C, which appears to be essentially exposure free in our semi-field experiment, suggests that these mosquitoes may have already fed when they entered the trap. These occasional specimens may have successful fed nearby and entered the ITT-C looking for either a second blood meal^27^ or shelter.

A pilot community-based surveillance system using ITT-B in urban Dar es Salaam has already proven to be representative, affordable, and effective in terms of mosquito catch and species composition.^19^ Crucially, it was also found to be three times less expensive than human landing catches per vector mosquito caught.^19^ The ITT-C appears to have all of these advantages and is more sensitive and protects the users. It may therefore be a useful sampling tool for routine monitoring of adult malaria-transmitting mosquitoes under programmatic conditions, such as those experienced by the Urban Malaria Control Program of Dar es Salaam.^4^,^5^,^28^

Any alternative mosquito sampling tool, apart from being safe and sensitive, must also yield epidemiologically representative estimates of human exposure to mosquito bites and pathogen transmission.^2^ Because the human landing catch technique is still believed to be the most reliable method for estimating the human biting rate,^3^,^29^–^31^ it may be necessary to validate the ITT-C by comparing it with this gold standard rather than the B design that preceded it. As previously suggested,^18^ we recommend that the ITT-C and other potentially useful methods be assessed in comparison with epidemiologic indicators of human infection so that the most meaningful entomologic approaches can be identified.

## Supplementary Material

Supplementary Figure

[Supplementary material]

## Figures and Tables

**Table 1:** Number of mosquitoes trapped by the B and C designs of the Ifakara Tent Trap*

Method	Trap nights	*Anopheles gambiae* s.l.			*Culex* spp.			*Mansonia* spp.		
		Total	Mean	Relative sensitivity	Total	Mean	Relative sensitivity	Total	Mean	Relative sensitivity
Ifakara C	20	714	35.7	2.1	350	17.5	2.0	774	38.7	1.8
Ifakara B	20	344	17.2	NA	174	8.7	NA	441	22.1	NA

* NA = not applicable because this is the reference method.

**Table 2:** Mosquito sampling sensitivity of the Ifakara Tent Trap model C compared with the B design and evaluated by using generalized estimating equations and expressed as the relative rate at which mosquitoes are caught*

Taxon	Trap type	RR (95% CI)	*P*
*Anopheles gambiae* s.l.			
	Ifakara C	1.92 (1.53–2.42)	< 0.001
	Ifakara B	1.00†	
*Culex* spp.			
	Ifakara C	1.90 (1.48–2.43)	< 0.001
	Ifakara B	1.00†	
*Mansonia* spp.			
	Ifakara C	2.30 (1.54–3.36)	< 0.001
	Ifakara B	1.00†	

* RR = relative rate; CI = confidence interval.

† Reference value.

**Table 3:** Influence of trapping method on the proportion of *Anopheles arabiensis* caught in the field and *An. gambiae* s.s. recaptured in the semi-field system that were fully or partly blood fed as determined by binary logistic regression*

Experiment	Trap type	Proportion fed (%)	OR (95% CI)	*P*
*An. arabiensis* in the field				
	Ifakara C	1.4 (10/703)	0.27 (0.12–0.60)	0.001
	Ifakara B	5.1 (17/336)	1.00†	NA
*An. gambiae* in the semi-field				
	Ifakara C	0.0 (0/190)	NE	NE
	Ifakara B	3.5 (6/171)	NE	NE

* OR = odds ratio; CI = confidence interval; NA = not applicable because this is the reference method; NE = not estimable.

† Reference value.
