# Supplementary figures and images for: An Exposure-Free Tool for Monitoring Adult Malaria Mosquito Populations
Source: Am J Trop Med Hyg. 2010 Sep;83(3):596–600. doi: 10.4269/ajtmh.2010.09-0682 (PMC2929057; doi:10.4269/ajtmh.2010.09-0682)

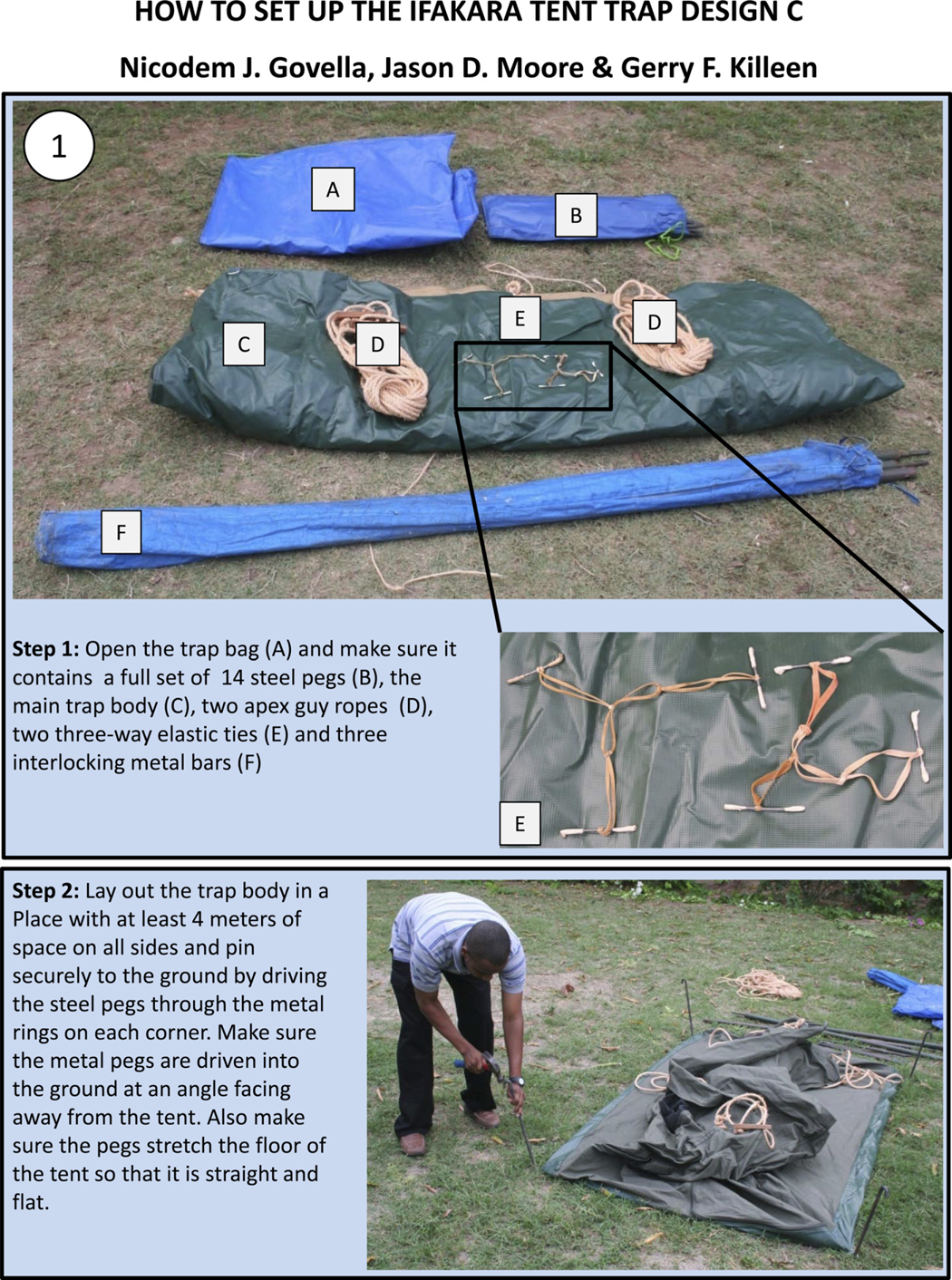

Supplement: Supplementary Figure [file SD2.tif]

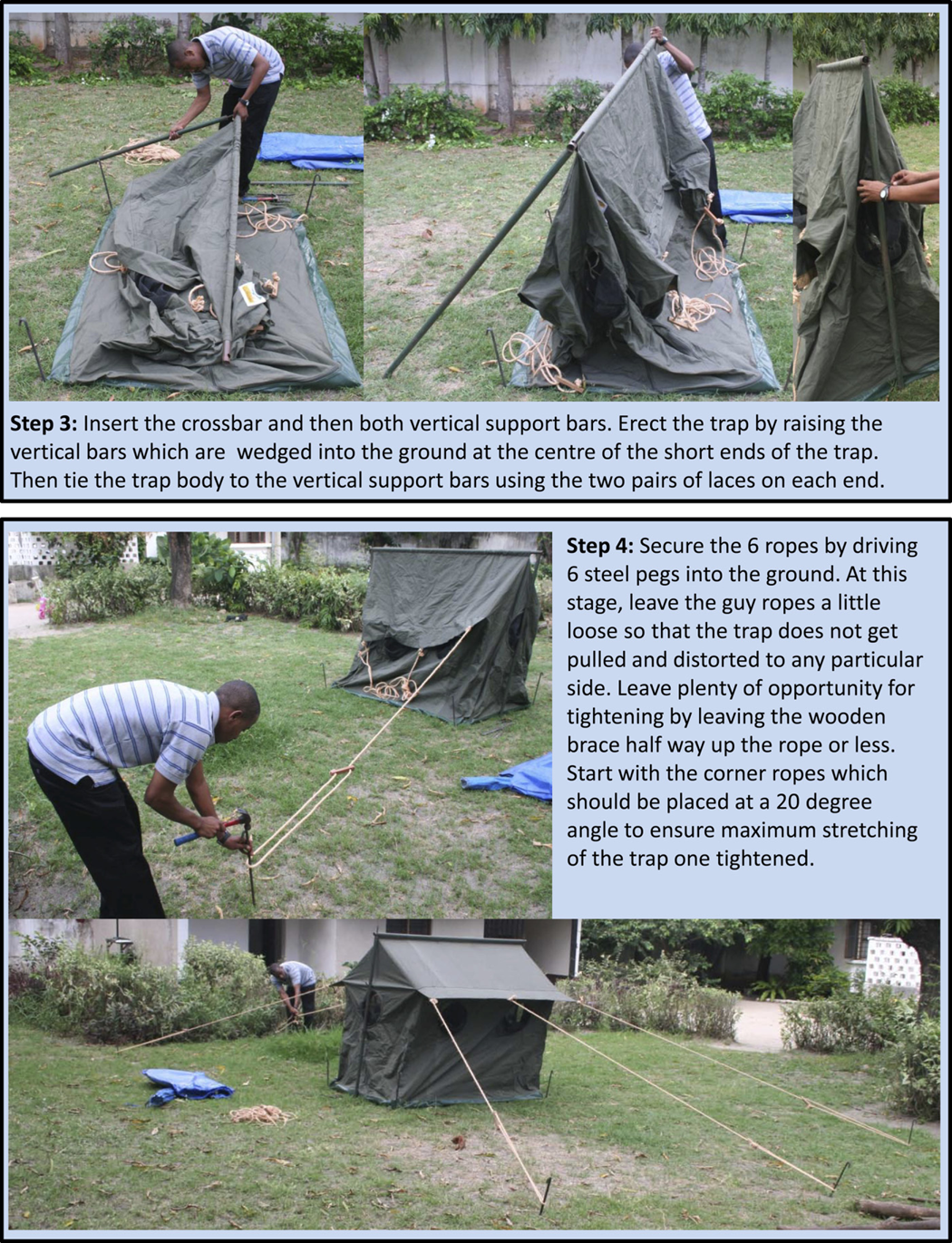

Supplement: Supplementary Figure [file SD3.tif]

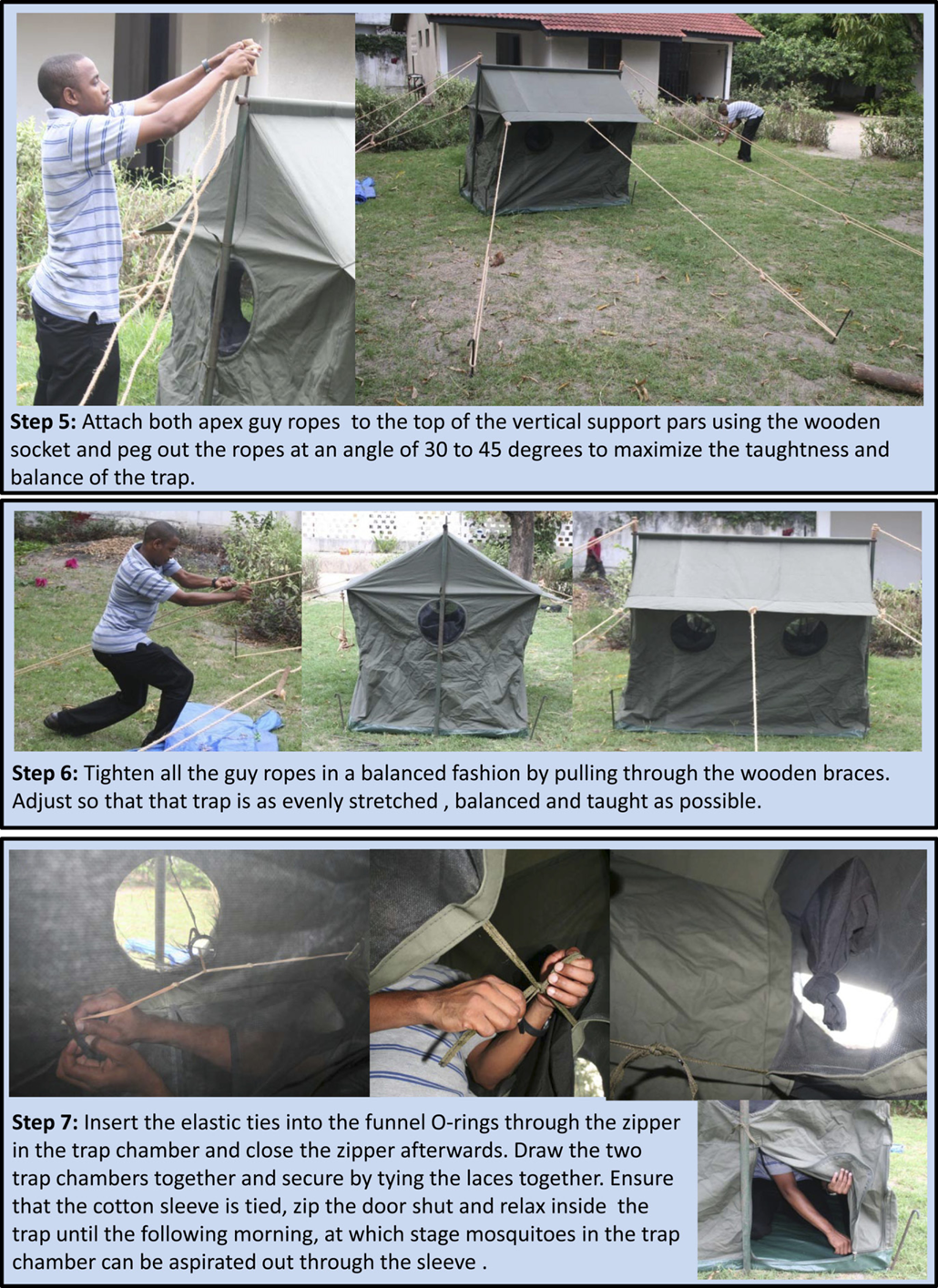

Supplement: Supplementary Figure [file SD4.tif]

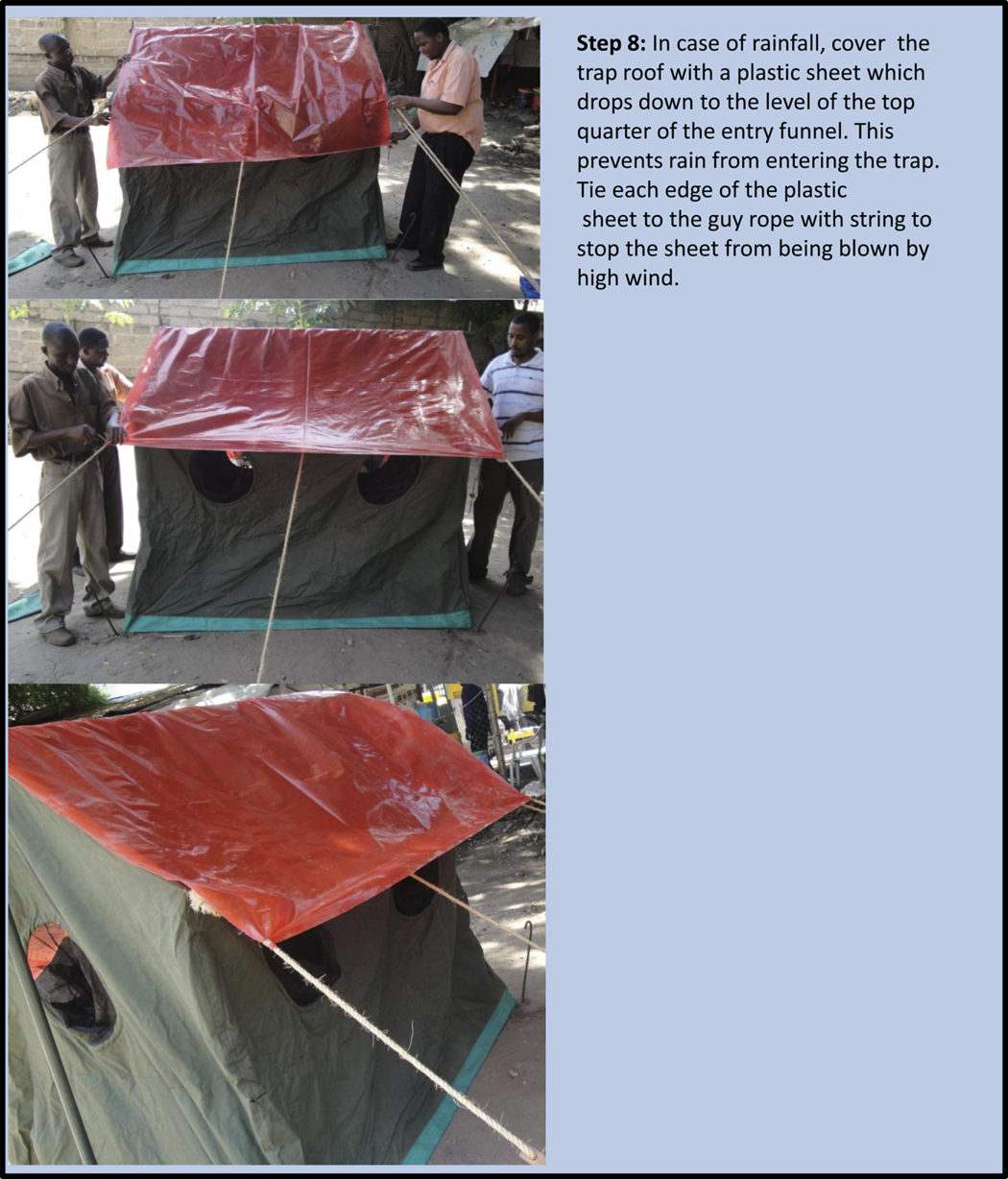

Supplement: Supplementary Figure [file SD5.tif]
