# Supplementary material for: An Exposure-Free Tool for Monitoring Adult Malaria Mosquito Populations
Source: Am J Trop Med Hyg. 2010 Sep;83(3):596–600. doi: 10.4269/ajtmh.2010.09-0682 (PMC2929057; doi:10.4269/ajtmh.2010.09-0682)
Supplement: [Supplementary material] [file supp_83_3_596__index.html]

 An Exposure-Free Tool for Monitoring Adult Malaria Mosquito Populations -- Govella et al. 83 (3): 596 Data Supplement - Supplementary material -- American Journal of Tropical Medicine and Hygiene **An Exposure-Free Tool for Monitoring Adult Malaria Mosquito Populations**  
 Am J Trop Med Hyg Govella et al. 83: 596

## Supplementary material

Tent trap setup instructions

**Files in this Data Supplement:**

- Supplementary material - Tent trap setup instructions
