# Supplementary material for: An Exposure-Free Tool for Monitoring Adult Malaria Mosquito Populations
Source: Am J Trop Med Hyg. 2010 Sep;83(3):596–600. doi: 10.4269/ajtmh.2010.09-0682 (PMC2929057; doi:10.4269/ajtmh.2010.09-0682)
Supplement: [Supplementary material] [file supp_83_3_596__1.pdf]

HOW TO SET UP THE IFAKARA TENT TRAP DESIGN C

Nicodem J. Govella, Jason D. Moore & Gerry F. Killeen

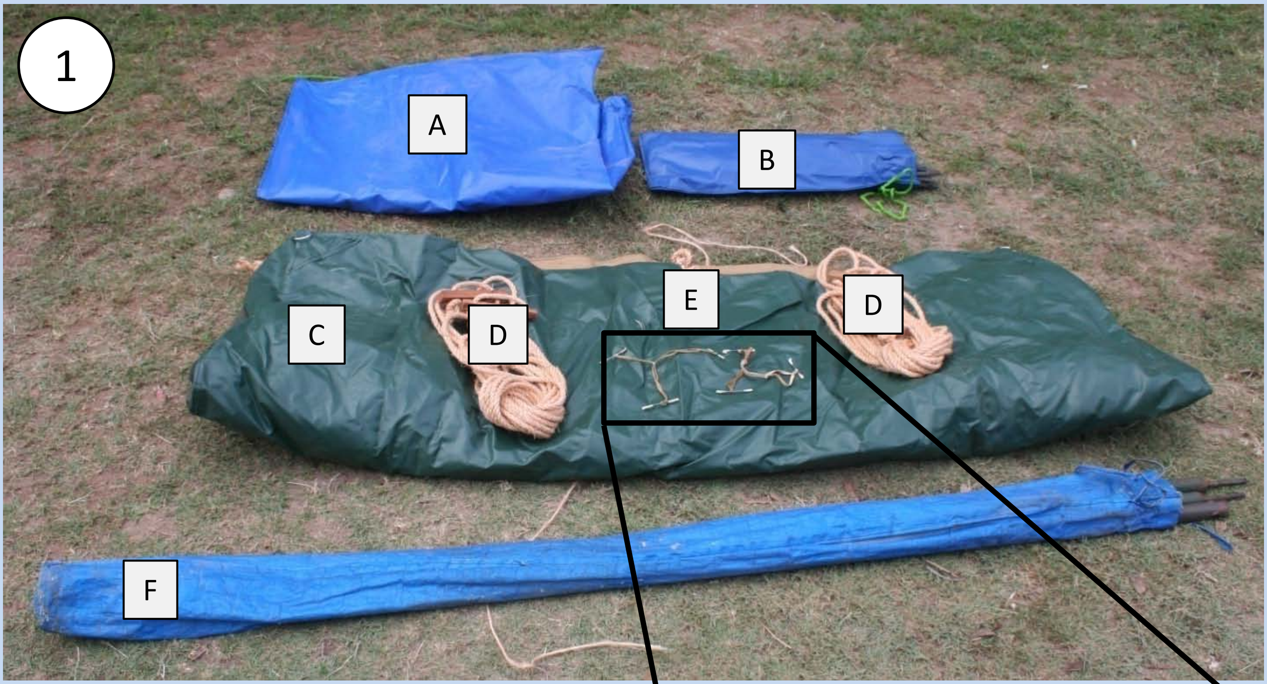

Step 1: Open the trap bag (A) and make sure it contains a full set of 14 steel pegs (B), the main trap body (C), two apex guy ropes (D), two three-way elastic ties (E) and three interlocking metal bars (F)

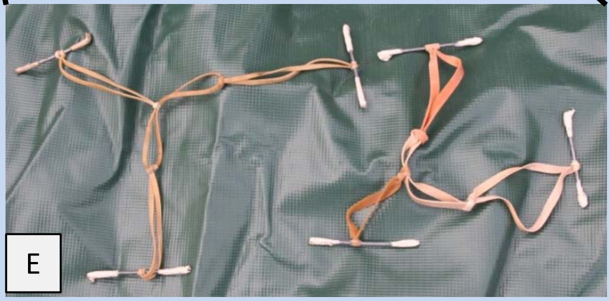

Step 2: Lay out the trap body in a Place with at least 4 meters of space on all sides and pin securely to the ground by driving the steel pegs through the metal rings on each corner. Make sure the metal pegs are driven into the ground at an angle facing away from the tent. Also make sure the pegs stretch the floor of the tent so that it is straight and flat.

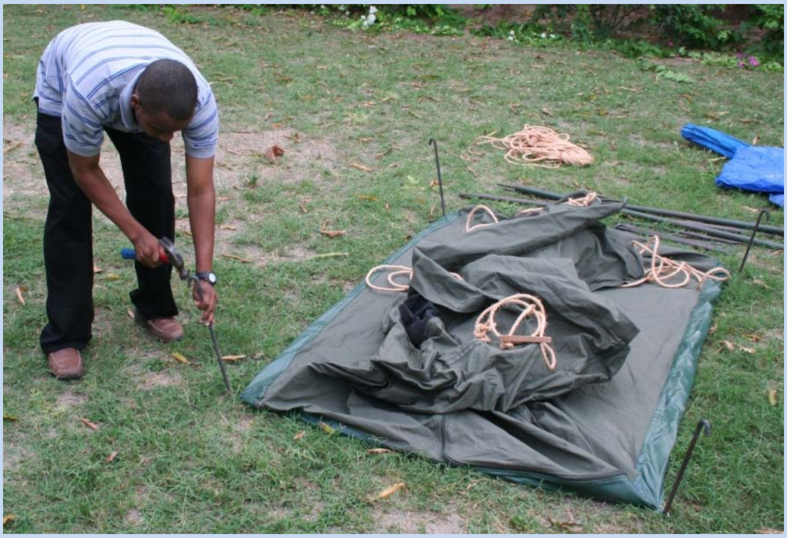

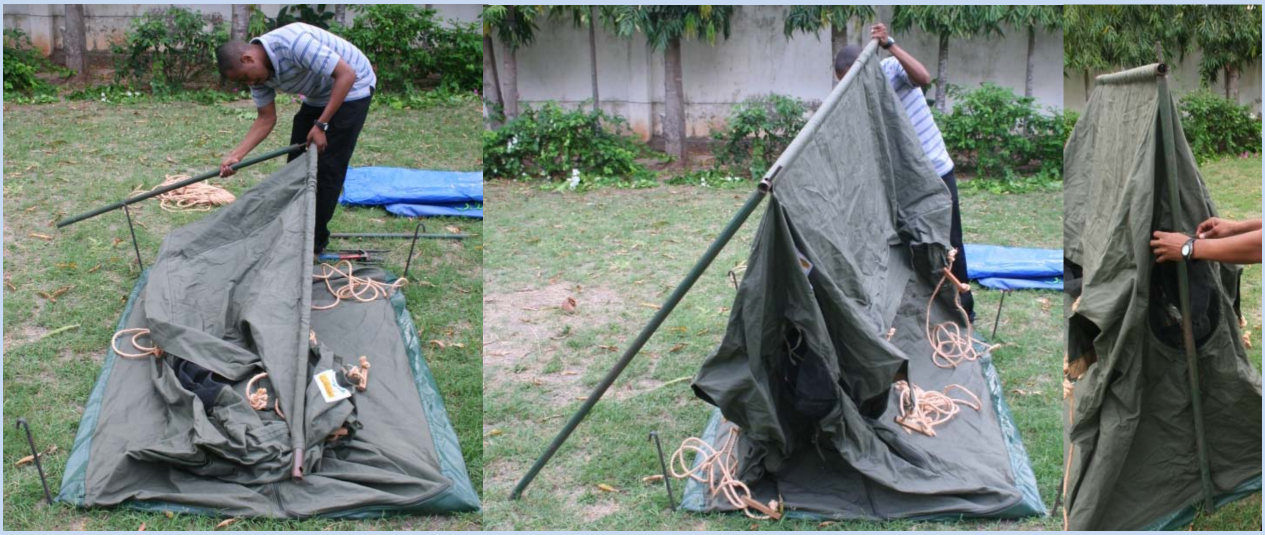

Step 3: Insert the crossbar and then both vertical support bars. Erect the trap by raising the vertical bars which are wedged into the ground at the centre of the short ends of the trap. Then tie the trap body to the vertical support bars using the two pairs of laces on each end.

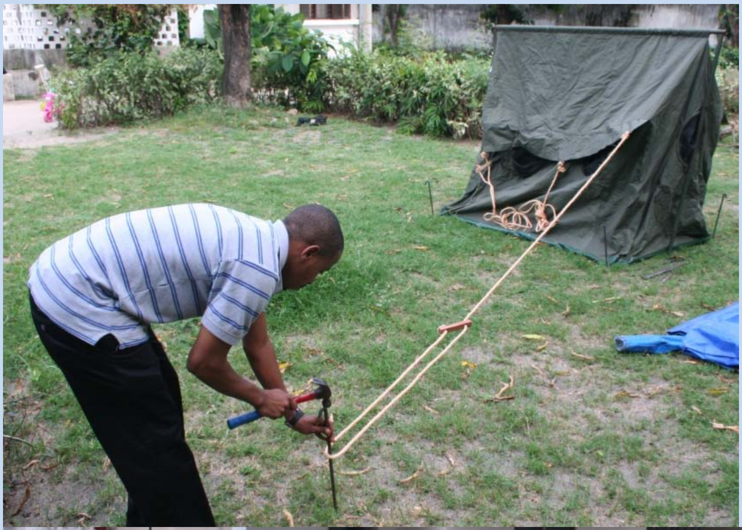

Step 4: Secure the 6 ropes by driving 6 steel pegs into the ground. At this stage, leave the guy ropes a little loose so that the trap does not get pulled and distorted to any particular side. Leave plenty of opportunity for tightening by leaving the wooden brace half way up the rope or less. Start with the corner ropes which should be placed at a 20 degree angle to ensure maximum stretching of the trap one tightened.

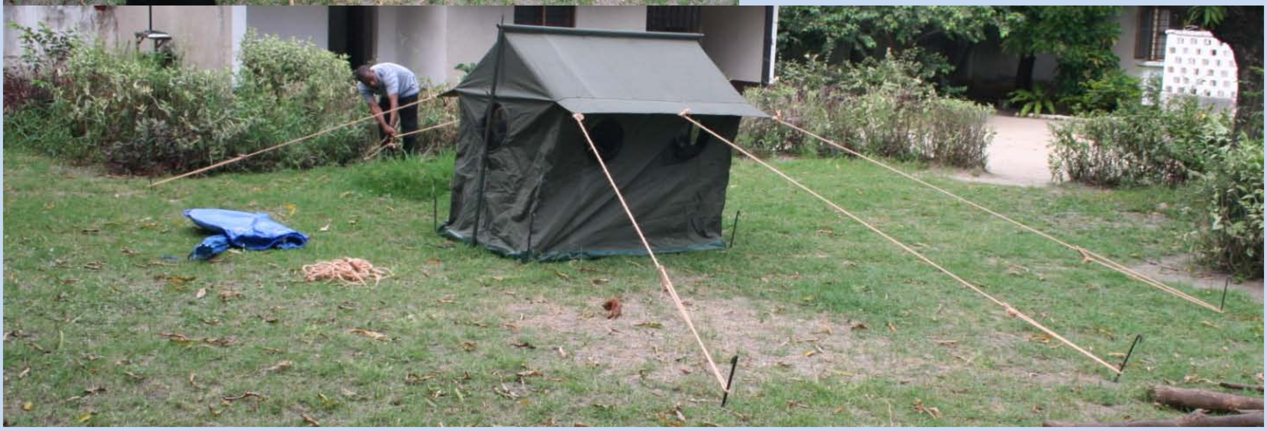

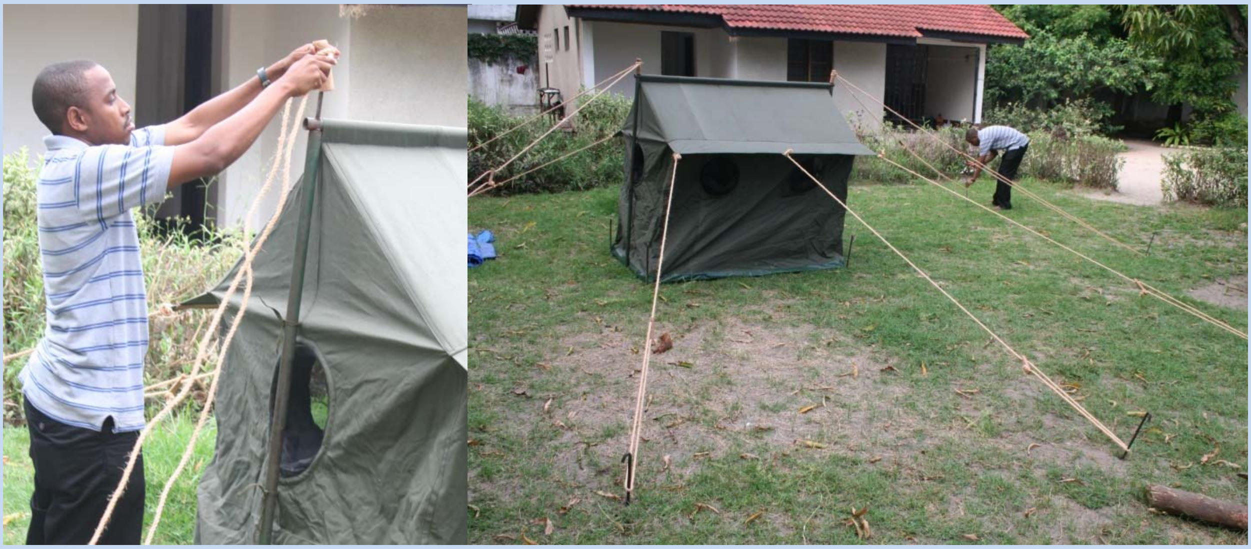

Step 5: Attach both apex guy ropes to the top of the vertical support pars using the wooden socket and peg out the ropes at an angle of 30 to 45 degrees to maximize the taughtness and balance of the trap.

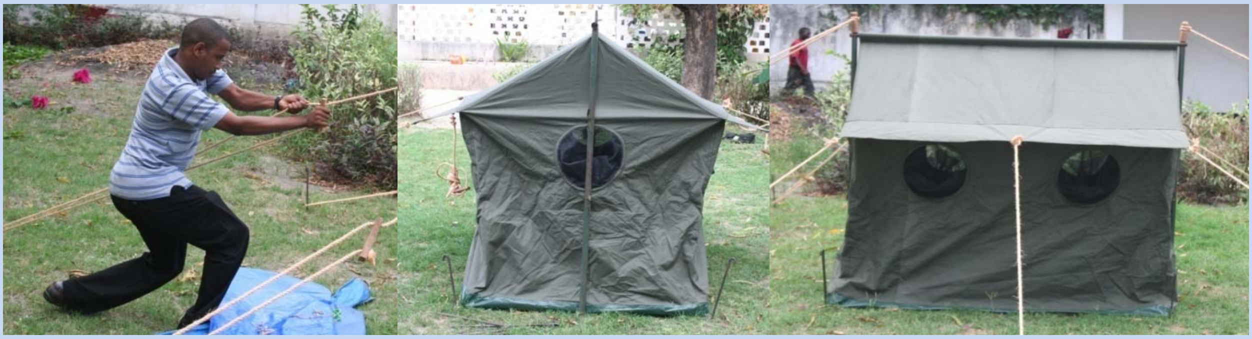

Step 6: Tighten all the guy ropes in a balanced fashion by pulling through the wooden braces. Adjust so that that trap is as evenly stretched , balanced and taught as possible.

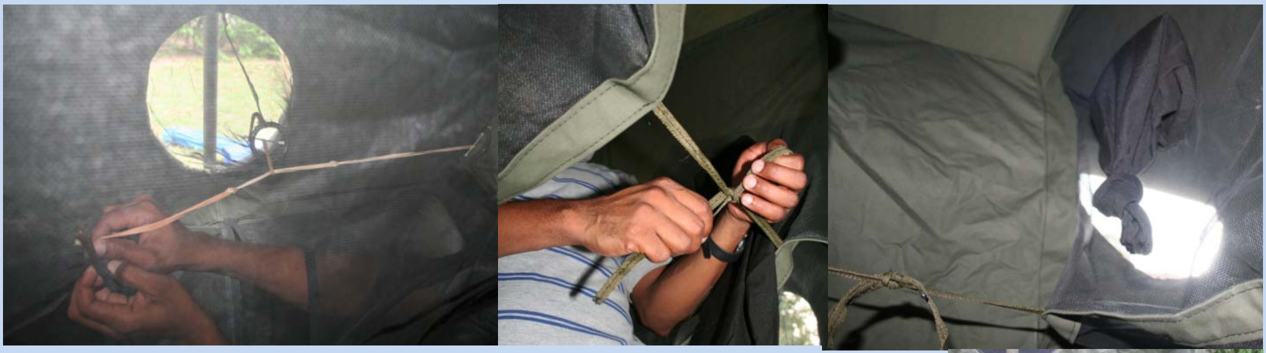

Step 7: Insert the elastic ties into the funnel O-rings through the zipper in the trap chamber and close the zipper afterwards. Draw the two trap chambers together and secure by tying the laces together. Ensure that the cotton sleeve is tied, zip the door shut and relax inside the trap until the following morning, at which stage mosquitoes in the trap chamber can be aspirated out through the sleeve .

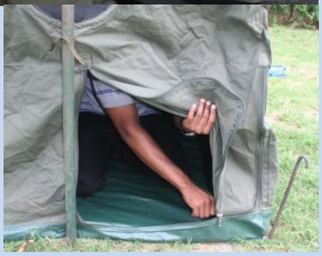

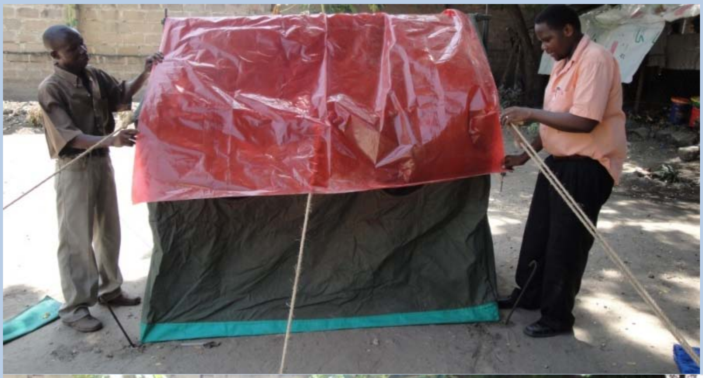

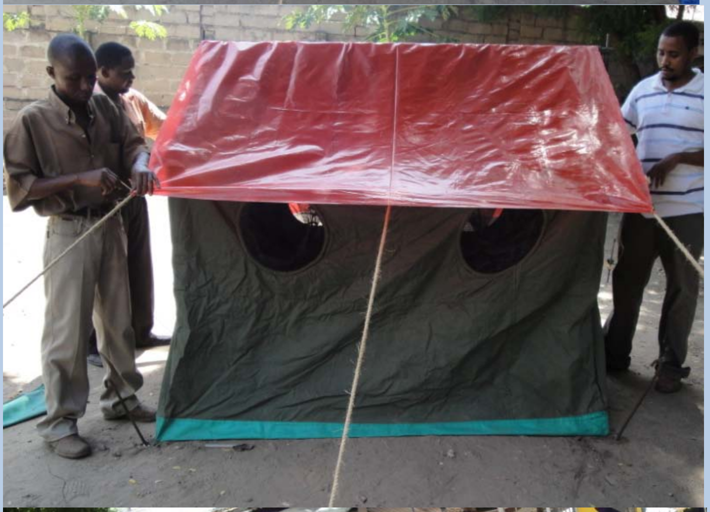

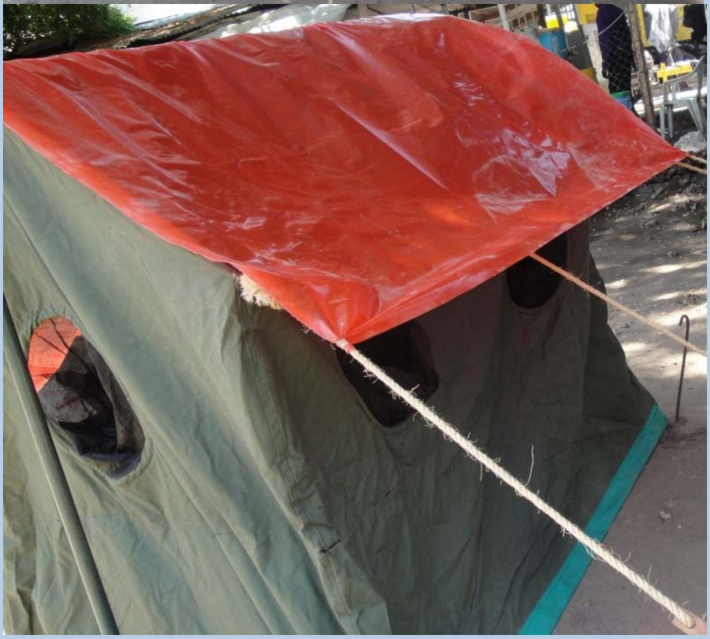

Step 8: In case of rainfall, cover the trap roof with a plastic sheet which drops down to the level of the top quarter of the entry funnel. This prevents rain from entering the trap. Tie each edge of the plastic sheet to the guy rope with string to stop the sheet from being blown by high wind.
